# Participation in a Prison-Based Training Programme Is Beneficial for Rescue Dogs

**DOI:** 10.3390/ani14111530

**Published:** 2024-05-22

**Authors:** Rebecca J. Leonardi, Sarah-Jane Vick, Hannah M. Buchanan-Smith

**Affiliations:** Psychology, Faculty of Natural Sciences, University of Stirling, Stirling FK9 4LA, UK; rebecca.leonardi@stir.ac.uk (R.J.L.);

**Keywords:** dogs, behaviour, welfare, positive reinforcement training, animal-assisted intervention, prison

## Abstract

**Simple Summary:**

There is an increasing number of dogs in rescue shelters, and they require rehoming. These dogs may have behavioural issues and lack training, both of which present barriers to their successful rehoming. There is evidence that prison-based dog training programmes may benefit those in custody as they learn skills and gain companionship. Less is known about how dog training programmes impact the dogs. We studied the behaviour and training performance of dogs in a prison-based programme for rescue dogs in which the prisoners find value in helping the dogs find permanent homes. Dogs attended the prison 1–3 times per week, under staff supervision and instruction. Our results showed that the rescue dogs had improved performance on training tasks. Their behaviour also changed; dogs rested or relaxed more in their kennels after the dog training programme than before it started, although other behaviours were not affected. Kennel staff rated the dogs’ behaviour as becoming more desirable and less undesirable after the dog training programme. However, some behaviour and welfare issues associated with relinquishment persisted. We conclude that prison-based dog training programmes can contribute to positive outcomes for dogs, as well as for humans, if conducted appropriately.

**Abstract:**

Dogs are often relinquished because of behavioural issues which may be exacerbated in rehoming centres. Prison-based dog training programmes (DTPs) may enhance outcomes for rescue dogs by providing socialisation and training opportunities to improve behaviour, welfare and likelihood of rehoming. We assessed whether dogs benefitted from participation, 1–3 times per week, in a prison-based DTP in which male young offenders learn how to train and care for dogs waiting to be rehomed. Within DTP sessions, there was significant improvement on a range of training tasks (n = 42 dogs). Analyses of videos (n = 17 dogs) in the kennels and a training barn pre- and post-DTP participation showed improvement in some positive behaviours, but no significant change in other behaviours. Subjective ratings by staff of the dogs’ behaviour were made (n = 20 dogs). Desirable behaviours (e.g., playful/friendly) increased, and most undesirable behaviours (e.g., frustrated and noisy) decreased. Participation in the DTP did not mitigate all negative behaviours. However, improvements are consistent with enhanced welfare and likelihood of successful rehoming. Prison-based DTPs can be effective in supporting the work of animal rescue organisations to improve outcomes for dogs, while offering people in custody an opportunity to engage in purposeful activity and provide a community service.

## 1. Introduction

Unprecedented numbers of unwanted dogs are relinquished to rescue organisations in the United Kingdom [1]. Dogs are commonly relinquished due to behavioural problems, such as aggression, hyperactivity, noisiness or a lack of training [2,3] and the shelter environment can exacerbate these problems [4,5,6]. In shelters, dogs can be subjected to the loss of attachment figures, unfamiliar people and routines, a lack of exercise, isolation, unpredictable noise, and a general loss of control over environmental contingencies [7]. Animal welfare is defined as an animal’s ability to adapt to and cope (positively and negatively) with the demands imposed by its environment [8], and incorporates physical health and mental and social well-being [9,10]. While there is considerable individual variation in dogs’ coping responses, the arousal or stress experienced in a shelter environment can impact their physiology, behaviour and welfare [5,11,12,13].

Human interaction and training can be effective in ameliorating the behavioural and physiological impact of relinquishment and enhancing the likelihood of adoption [11,14,15,16,17,18]. The method of training used is important; positive reinforcement is shown to improve human–dog relationships, while punishment elicits anxiety and impairs welfare over time [19,20]. While animal rescue organisations strive for shelter provisions to promote optimal welfare, time and staffing resources can limit their capacity to meet the needs of individual dogs [21]. Prison-based dog training programmes (DTPs) are an increasingly popular model for supporting rescue organisations, by increasing the capacity to engage rescue dogs in extensive training and socialisation to promote positive outcomes [22,23,24].

Unlike most forms of animal-assisted intervention (AAI), which aim to benefit people, DTPs are intended to be mutually beneficial for people and dogs [25]. For incarcerated individuals, DTPs provide an opportunity to make a positive contribution to society and are associated with improvements in psychological well-being, social competencies, institutional behaviour and reduced recidivism [26,27]. For DTPs working with rescue dogs, the primary aim is to improve the dogs’ behaviour and subsequently the chances of being successfully rehomed [23,25]. However, there has been surprisingly limited evaluation of the welfare implications and outcomes for the dogs involved in DTPs [24] or within AAIs more broadly [20,28].

When engaging animals in any AAI there is an ethical responsibility to optimise welfare provisions [28,29]. From One Health and One Welfare perspectives, AAIs should both improve health and wellbeing for humans and provide a positive experience for the animals involved [30,31]. Dogs’ adaptability and their capacity to facilitate positive interactions with people makes them highly suitable to engage in many types of AAI [20,32]. Individual dogs should be carefully assessed for their suitability to engage in an AAI, based on their behaviour, temperament and preferences. Despite careful selection there are many potential stressors in AAIs, including inconsistent handling or training, interaction with strangers, and the inhibition of normal behaviours [33], and therefore, the evaluation of dog welfare is essential.

Reviews of canine welfare within AAIs highlight the limited evidence available and inconsistent findings, in terms of detecting changes in behavioural, physiological and subjective measures, which are likely to reflect both the heterogeneity of AAIs and variability in the individual dog’s responses in this context [20,28,34]. Overall, participation in AAIs is likely to be stimulating for many dogs and should not be overly stressful, given a careful selection of dog-handler dyads and careful monitoring of an individual dog’s responses [28,33]. Although DTPs specifically aim to benefit rescue dogs, there is a lack of evidence regarding their efficacy and potential welfare implications [24]. Hennessy et al. [23] concluded that rescue dogs benefited from participation in a 3-week DTP, in which dogs lived with their trainers, compared to dogs who remained at the shelter. For dogs in the DTP, but not the control condition, training performance increased and behavioural arousal in an unfamiliar situation decreased. However, physiological measures did not differ between these groups; plasma cortisol levels were stable but pituitary hormone (ACHT) increased in both groups over time [23], consistent with evidence that stress experienced in a shelter environment can lead to dysregulation in the hypothalamic–pituitary–adrenal axis in dogs [12].

In this study, we evaluate whether rescue dogs benefitted from their participation in a DTP, in which male young offenders in a Scottish prison provide opportunities 1–3 times per week for training and socialisation (see [35]). The dogs’ behaviour and welfare are assessed before and after participation in a DTP in terms of training performance within sessions, behavioural observations in the rehoming centre, and subjective ratings of behavioural responses and welfare across a range of contexts. In line with the aims of the DTP, we predicted that participation would improve rescue dogs’ training performance and that the provision of training and socialisation opportunities would result in improved behaviour and welfare beyond the training context, contributing to an increased likelihood of adoption [15,23].

## 2. Materials and Methods

### 2.1. Ethical Statement

The study complies with guidelines on conducting ethical research with animals [36]. The selection of dogs for participation in the DTP adheres to IAHAIO guidance [29] that all animals participating in AAIs, should be assessed by an animal behaviourist for their suitability and be ‘in good health, both physically and emotionally’ (p. 7).

### 2.2. Study Participants

Dogs (n = 52) were selected for participation in the DTP from two Dogs Trust Rehoming Centres close to the prison: West Calder (June–October 2013), and subsequently, their Glasgow Rehoming Centre (November 2013–June 2014). Individual dogs were assessed for suitability by the course instructor (RJL) and rehoming centre staff after a minimum of 1 week in the centre, allowing staff to become familiar with the dogs, and for the dogs to adjust to a new environment [37,38]. Selection was based on good health and the identification of potential barriers to rehoming which could reasonably be expected to be modified through increased training and socialisation, such as lacking confidence or life skills, or being overly reactive or excitable. Dogs attended the DTP for a median duration of 7 weeks (range 1–23) and 85% were happily withdrawn from the DTP due to rehoming (n = 44/52). Eight were withdrawn due to welfare concerns (travel or anxiety issues, n = 3) or logistical reasons (change of rehoming centre used, n = 4; dog transferal, n = 1). Dogs not rehomed during their first course could continue to a second (n = 19) or third DTP course (n = 6), following an interval of approximately 1 month.

### 2.3. Paws for Progress DTP

**Figure 1:**
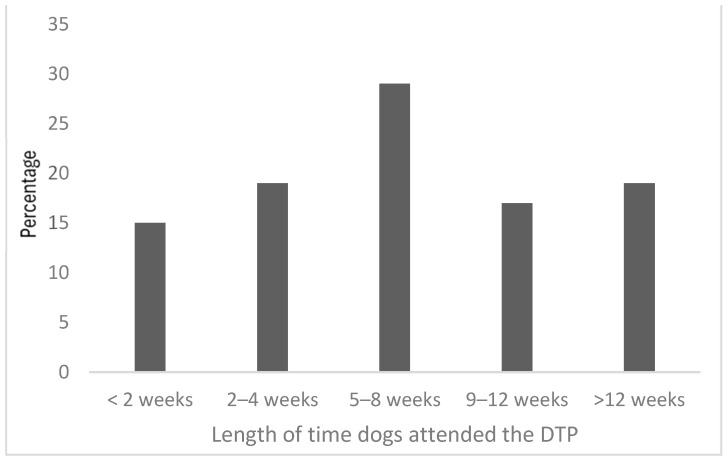
The percentage of dogs (n = 52) that attended the DTP by the number of weeks.

Dogs attended between 1–3 sessions per week, over an 8-week course. Sessions were approximately 2.5 h in duration, in addition to a 30 min return journey from the shelter to the prison. Typically, 4–6 dogs attended a session and each had a designated student handler, supported by up to 5 peer mentors (also in custody) and under the supervision of three staff, including at least one experienced dog trainer. Each session began with a short walk and outdoor training activities, followed by feeding, down time for rest and interaction, and training indoors. The outdoor area comprised a grassed and concrete area, allowing dogs to train at a reasonable distance (i.e., 10–30 m if required) from other dogs. When inside, each dog had a designated area with barriers to prevent visual access to other dogs during rest periods and individual training. The length of time the dogs attended the DTP is shown in Figure 1.

### 2.4. Methodology

#### 2.4.1. Training Progress

The dogs’ responses to training tasks were assessed weekly by their designated student handler, in collaboration with DTP staff members. Nineteen training tasks were selected based on the Association of Pet Dog Trainers’ Good Companion Award assessment (APDT; https://apdt.co.uk/good-companion-awards/ accessed on 6 May 2024). Training performance was scored using a 5-point Likert scale: 0 = no appropriate response, 1 = difficult to achieve, 2 = manageable with repeated guidance, 3 = accomplished with minimum guidance, 4 = immediate response. Discrepancies between handler and staff scores were minor and infrequent, with agreement reached through a discussion of the dog’s specific responses on the relevant tasks.

#### 2.4.2. Behaviour Observations

Video observations were collected for 24 dogs housed at the Dogs Trust Glasgow rehoming centre (February–June 2014). A Sony camcorder and tripod were used to record the dogs every two weeks on non-DTP days, at consistent times in relation to their daily routine, and in both their home kennel and a training barn. Each kennel (3 m × 2 m) had beds and water available and allowed access to an outdoor area. For filming purposes, each dog’s usual conditions were maintained (e.g., presence of a kennel mate) but outdoor access was temporarily restricted. The training barn was a large indoor area (approx. 20 m × 30 m) where dogs were free to move around, with two toys placed on the floor. At each time point, individual dogs were filmed in the presence of the same familiar staff member, seated in the middle of the barn, who remained passive unless the dog actively solicited interaction and ceased to engage if a dog became over excited (following [39]).

All dogs were initially observed 1 week prior to joining the DTP to provide a baseline (pre-DTP) for behaviour in both kennel and training barn. Videos were approximately 3.5 min in duration and the middle 3 min were coded using Cowlog (version 2.11; [40]). Point sampling at 10 s intervals was used to record the dogs’ location and behaviour (see Table 1 and Table S1). Behaviours were allocated to one of four mutually exclusive categories: Positive Active (play with toy, alert or explore, also includes playful/friendly in the training barn), Positive Inactive (resting or relaxed), Negative Active (destructive, excited, frustrated or reactive) and Negative Anxious (repetitive, stressed or vigilant). Two context-specific behaviours were also coded at each point sampling interval; location in kennel (front or rear) and interaction (either in contact or visual attention) with the person present in the training barn. A single observer coded all videos, with time points of pre- (baseline) or post-DTP (the final session recorded) blinded, and two videos in each environment were also coded by a second observer; both intra- (r = 0.96), and inter-observer reliability (r = 0.94) were well above acceptable levels of 0.7 [41].

#### 2.4.3. Subjective Ratings

Staff at the Dogs Trust Glasgow Rehoming Centre rated the overall behaviour and welfare for 24 dogs (February–June 2014). Those staff completing ratings were familiar with the dogs and scored the same dogs weekly, with relevant input from other members of the staff team if required. The contexts, behaviours and definitions included in the assessment were generated in collaboration with the team of raters, an approach resembling Free Choice Profiling (FCP) methods and effective for qualitative behaviour assessments in dogs [39]. Staff were asked to focus on the dogs’ interactions with their environment and to describe their expressive demeanour across four contexts: when the dog was in their kennel, on a walk, passing another dog or interacting with people. Nine behaviours were rated across all four contexts (see Table 1 and Table S1): two were considered Desirable (playful/friendly and relaxed) and seven Undesirable (excitable, reactive, noisy, frustrated, subdued/depressed, stressed and vigilant). Five context-specific behaviours were also rated: destructive and repetitive behaviours in the kennel, avoidant behaviour when not in the kennel, pulling on the lead during walks, and pulling toward either when passing other dogs or people. Staff rated the extent to which each dog displayed each behaviour over the preceding week using a Likert scale: 1 = not at all; 2 = not very often; 3 = sometimes; 4 = quite often; and 5 = very often.

### 2.5. Data Analysis

Data were entered and analysed using IBM SPSS (version 25) using non-parametric tests; training and rating data were ordinal, and almost all of the behavioural measures were not normally distributed.

#### 2.5.1. Training Progress

Only those dogs who had training scores collected over 3 or more weeks within the same course were retained in the analysis (n = 42 dogs). A total weekly training score was calculated and converted into a percentage of the maximum score possible (out of 76). Wilcoxon tests were used to compare total training percentage scores in the first and last session attended, for both the first and second (n = 19) course attended (only 6 dogs attended for a third course). To examine progress during the first 3 weeks in the DTP, overall percentage and scores on each task were compared using Friedman’s tests, with pairwise comparisons performed using Wilcoxon’s tests (with Bonferroni correction at *p* < 0.001). An assessment of criteria for meeting the APDT Good Companion Award was made for dogs attending the DTP for at least one month (n = 34 dogs). Finally, because the duration of participation in the DTP was variable, the relationships between the total time spent in the DTP (in weeks) and both the initial training score and the change in total training scores (between the first and final session recorded) were explored using Spearman’s rank correlations.

#### 2.5.2. Behaviour Observations

Videos were collected of 24 dogs in both their kennel and in a training barn; seven dogs were excluded because they were either rehomed within 4 weeks or spent more than two-thirds of an observation period out of view, leaving a sample of 17 dog videos for analyses. Behaviours were categorised as Positive Active, Positive Inactive, Negative Active and Anxious; an estimated percentage of time engaged in each behavioural category was calculated and adjusted for time spent out of view. The estimated percentage of time spent in the front half of the kennel, and the total amount of interaction (either in contact or visual attention) with the person present in the training barn were also calculated. Wilcoxon’s tests were used to compare behaviour in the baseline and final observations in each environment. Spearman’s correlations were used to test for an association between total time spent in the DTP (in weeks) and both baseline levels and change observed over time (difference between baseline and final session, positive values indicate an increase from baseline) for each behavioural category.

#### 2.5.3. Subjective Ratings

Only those dogs with completed staff ratings available across at least a 3-week period were retained in the analyses (n = 20). The perceived prevalence of Desirable and Undesirable behaviours (calculated as a percentage of the total possible score) were compared between baseline and final ratings in the DTP using Wilcoxon comparisons, both overall and for each of the four contexts (kennel, on a walk, passing another dog or interacting with people) in which the dogs were evaluated. The percentage scores for individual behaviours were also compared between baseline and final ratings. Spearman’s correlations are used to test for an association between time spent in the DTP (in weeks) and baseline Desirable and Undesirable ratings, and with the percentage change in these scores (positive values indicate an increased prevalence from baseline).

## 3. Results

### 3.1. Training Progress

Total training scores improved between the first (median = 44, IQR 39–53) and last session attended (median = 77, IQR 70–94; *z* = 5.65, n = 42, *p* < 0.001, η^2^ = 0.79). Significant progress was evident within the first 3 weeks (*χ*^2^ = 76.06, df = 2, n = 42, *p* < 0.001, η^2^ = 0.87) with higher scores on 14/19 training tasks achieved by the second week (Table 2). All dogs had higher total training scores within 3 sessions and most dogs (32/34) attending the DTP for at least 1 month gained an APDT Good Companion Award. For dogs attending a second course, total percentage scores increased between the first (median = 64, IQR 55–84) and last session attended (median = 86, IQR 79–99; *z* = 3.76, n = 19, *p* < 0.001, η^2^ = 0.75). Total time spent in the DTP was not correlated with training scores recorded in the first session (*r_s_* = 0.21, n = 42, *p =* 0.19) but was positively correlated with the total improvement in scores between the first and final session (*r_s_* = 0.548, n = 42, *p* < 0.001).

### 3.2. Behaviour Observations

**Figure 2:**
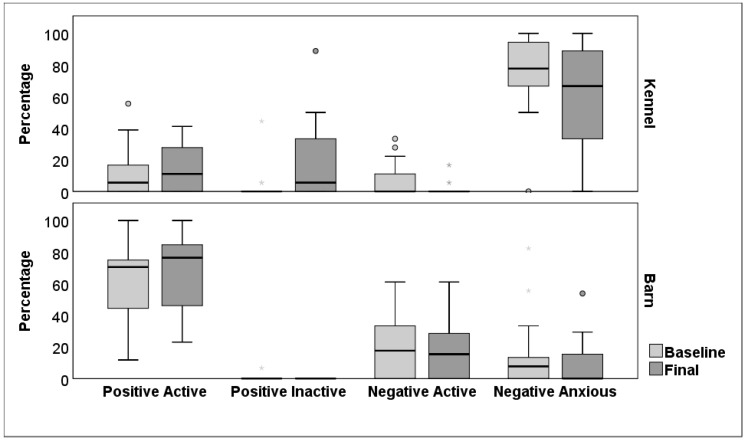
Estimated median (inter-quartile range) percentage of time spent in Positive Active, Positive Inactive, Negative Active and Negative Anxious behaviours during baseline (pre-DTP) and final observations following DTP participation in home kennel and training barn (n = 17 dogs). Central bar is the median, box indicates the upper and lower quartile for the middle 50%, and whisker represents the upper and lower 25%. Asterisks and circles show outliers.

In their home kennel, the dogs spent the majority of time engaging in Negative Anxious behaviours, with no significant difference between baseline (median = 78, IQR 64–97) and final observations (median = 67, IQR 33–91; *z* = 1.51, n = 17, *p* = 0.14, η^2^ = 0.067; Figure 2). There was also no significant change in either Positive Active (baseline median = 6, IQR 0–25, final median = 11, IQR 0–28; *z* = 0.68, n = 17, *p* = 0.50; η^2^ = 0.01) or Negative Active behaviours (baseline median = 0, IQR 0–11, final median and IQR = 0; *z* = 1.69, n = 17, *p* = 0.09, η^2^ = 0.08). However, there was an increase in Positive Inactive behaviours between baseline (median and IQR = 0) and final observations (median = 6, IQR 0–39; *z* = 2.10, n = 17, *p* = 0.04, η^2^ = 0.13). The amount of time the dogs spent in the front half of their kennel did not significantly increase between baseline (median = 50, IQR 16–86) and final observations (median = 83, IQR 33–92; *z* = 1.17, n = 17, *p* = 0.24, *η*^2^ = 0.004).

Observations in the training barn demonstrated that the dogs spent most of their time engaged in Positive Active behaviours, with no significant difference between baseline (median = 71, IQR 42–76) and final observations (median = 76, IQR 45–85; *z* = 0.59, n = 17, *p* = 0.55, η^2^ = 0.001; Figure 2). Positive Inactive behaviours were rare and did not differ between observations (all medians and IQR = 0; *z* = 1, *p* = 0.32, n = 17, η^2^ = 0.03), neither did Negative Active (baseline median = 18, IQR 0–33, final median = 15, IQR 0–32; z = 0.39, n = 17, *p* = 0.70, η^2^ = 0.01) or Negative Anxious behaviours (baseline median = 8, IQR 0–17, final median = 0, IQR 0–17; *z* = 0.74, n = 17, *p* =0.46, η^2^ = 0.01). There was no significant difference in the amount of social interaction with the staff member present (baseline median = 18, IQR 6–53; final median = 33, IQR 17–56; *z* = 0.70, n = 17, *p* = 0.49, η^2^ = 0.01).

The total length of time spent in the DTP was positively correlated with the baseline level of Negative Active behaviour observed in the home kennel (r = 0.57, n = 17, *p* = 0.02) and associated with larger reductions in Negative Active behaviour from baseline levels (*r_s_* = −0.57, n = 17, *p* = 0.02). There were no other significant relationships between the length of time spent in the DTP and either baseline levels or changes in behavioural profiles observed in either home kennels or training barn (all *p*s > 0.05; Table 3).

### 3.3. Subjective Ratings

**Figure 3:**
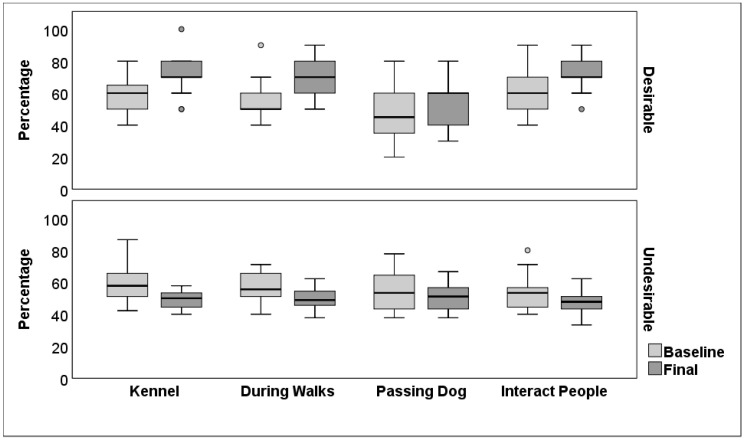
Median (inter-quartile range) percentage of subjective ratings of Desirable and Undesirable behaviours during baseline (pre-DTP) and final observations following DTP participation across four contexts (n = 20 dogs). Central bar is median, box indicates the upper and lower quartile for the middle 50%, and whisker indicates the upper and lower 25%. Circles show outliers.

Subjective ratings of the prevalence of Desirable behaviours increased between baseline (median = 57, IQR 48–63) and final ratings (median = 66, IQR 58–74; *z* = 3.28, n = 20, *p* = 0.001, *η*^2^ = 0.27). There was an increase in both playful/friendly (*z* = 3.09, *p* = 0.002, *η*^2^ = 0.24) and relaxed behaviours (*z* = 2.94, *p* = 0.003, *η*^2^ = 0.22; Table 4). Desirable behaviours were considered more prevalent in 90% (18/20) of dogs following the DTP. In terms of context, ratings of Desirable behaviours increased between baseline and final ratings in the home kennel (baseline median = 60, IQR 50–70; final median = 70, IQR 68–80; *z* = 3.34, n = 20, *p* = 0.001, *η*^2^ = 0.28), on walks (baseline median = 55, IQR 50–60; final median = 70, IQR 60–80; *z* = 3.58, n = 20, *p* < 0.001, *η*^2^ = 0.32) and during interactions with people (baseline median = 60, IQR = 50–70; final median = 70, IQR 70–83; *z* = 2.97, n = 20, *p* = 0.005, *η*^2^ = 0.22), but not in relation to passing other dogs (baseline median = 55, IQR 38–60; final median = 60, IQR 40–60; *z* = 1.31, n = 20, *p* = 0.19; *η*^2^ = 0.04; Figure 3).

**Figure 4:**
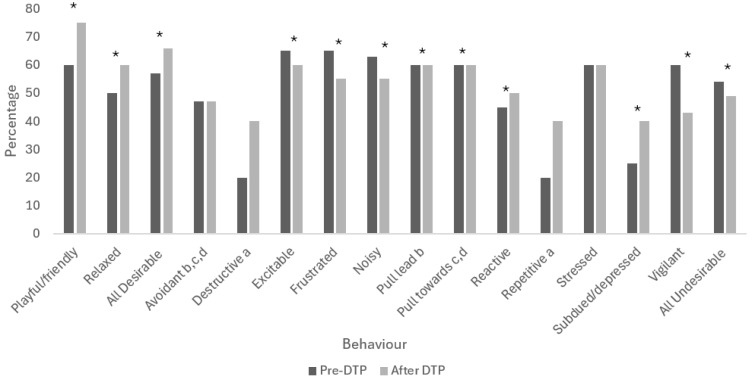
Median percentage scores for behavioural categories rated by staff at baseline (pre-DTP) and the final ratings after DTP participation (n = 20 dogs). Rating scores ranged from 1 = not at all to 5 = very often (see Section 2.4.3). Asterisks indicate significant differences (*p* < 0.05); inter-quartile ranges and statistical output are found in Table 4.

In contrast, Undesirable behaviours were considered less prevalent following participation in the DTP (median = 49, IQR 43–54) than at baseline (median = 54, IQR 48–61; *z* = 2.89, *p* = 0.004, *η*^2^ = 0.21). The overall prevalence of Undesirable behaviours was lower for 70% (14/20) of dogs following the DTP. Specifically, the dogs were judged as being less excitable (*z* = 2.43, *p* = 0.02, *η*^2^ = 0.15), frustrated (*z* = 2.62, *p =* 0.01, *η*^2^ = 0.172), noisy (*z* = 2.99, *p* = 0.003, *η*^2^ = 0.22), reactive (*z* = 2.07, *p* = 0.04, *η*^2^ = 0.11) and vigilant (*z* = 3.11, *p =* 0.002, *η*^2^ =0.24), and less likely to pull on their lead (*z* = 2.271, *p* = 0.02, *η^2^* = 0.13), or to pull towards other dogs or people (*z* = 2.50, *p* = 0.01, *η*^2^ = 0.16; Figure 4, Table 4). There was no significant change in avoidant (*z* = 0.37, *p* = 0.71, *η*^2^ = 0.003) and stressed behaviours (*z* = 1.66, *p* = 0.10, *η*^2^ = 0.07), or in destructive (*z* = 0.61, *p* = 0.54, *η*^2^ = 0.01) and repetitive (*z* = 0.28, *p* = 0.78, *η*^2^ = 0.002) behaviours in kennels. However, there was a significant increase in ratings of subdued/depressed behaviour (*z* = 2.01, *p* = 0.04, *η*^2^ = 0.10). In relation to context, the overall prevalence of Undesirable behaviours decreased in kennels (baseline median = 58, IQR 51–65; final median = 51, IQR 44–53; *z* = 3.30, n = 20, *p* = 0.001, *η*^2^ = 0.27), on walks (baseline median = 54, IQR 51–65; final median = 50, IQR 46–56; *z* = 3.13, n = 20, *p* = 0.002, *η*^2^ = 0.25) and during interactions with people (baseline median = 53, IQR 44–56; final median = 49, IQR 44–52; *z* = 2.79, n = 20, *p* = 0.02, *η*^2^ = 0.20), but not in relation to passing other dogs (baseline median = 53, IQR 44–63; final median = 51, IQR 44–56; *z* = 1.14, n = 20, *p* = 0.26, *η*^2^ = 0.03; Figure 3).

Total time in weeks spent in the DTP was negatively correlated with baseline ratings of Desirable behaviours (*r_s_* = −0.45, n = 20, *p* = 0.046) and positively correlated with baseline ratings of Undesirable behaviours (*r_s_* = 0.47, n = 20, *p* = 0.035). However, the total time spent in the DTP was not significantly correlated with the overall amount of change in ratings for either Desirable (*r_s_* = 0.26, n = 20, *p* = 0.27) nor Undesirable behaviours (*r_s_* = −0.29, n = 20, *p* = 0.22).

## 4. Discussion

Within a few sessions, there was significant improvement on a range of training tasks that aim to help the dogs interact positively with people and be more manageable in the home environment and on walks, as these are considered important in enhancing the likelihood of successful adoption [15,23,42]. While training improvements were quickly achieved, progress continued; the overall change in training score, but not baseline score, was positively correlated with total time spent on the DTP. In addition to improved performance on tasks, the predictability of these interactions and the sense of control facilitated by positive reinforcement training methods may also serve to reduce stress and improve welfare during sessions [43,44].

The provision of training and socialisation within the DTP was expected to lead to improved behaviour and welfare beyond the training context [15,23]. Training and socialisation are associated with a decrease in problematic behaviour and anxiety in dogs [45,46], and dogs that have not been involved with any professional training are reported to be less calm, less trainable and less sociable [47]. Training and socialisation opportunities can contribute to an increased likelihood of adoption. For example, Luescher and Medlock [15] reported that obedience training (walking on a lead, sitting, not barking and staying at the front of the kennel) at the shelter considerably improved subsequent adoption rates compared to dogs in an environmental enrichment control group. Behaviour is an important factor in adoption choices and the success of adoption; potential adopters state preferences for dogs who are playful, energetic, friendly, calm, attentive and responsive but not inattentive, not social, or too active or energetic [48]. Problematic behaviours are perceived to be indicators of compromised welfare and can subsequently be a long-term cause of distress, to both the owner and the dog [49].

Brief video observations indicated a limited impact of DTP participation on the prevalence of positive and negative behaviour at the rehoming centre; there was considerable individual variation in behavioural profiles which also differed between home kennels and training barn. The dogs spent more time engaged in Positive Inactive behaviours (e.g., relaxed or resting) in kennels following DTP participation. Baseline levels of Positive Inactive behaviour were not correlated with subsequent time in the DTP, which is consistent with previous evidence that potential adopters often prefer more active dogs [48,50]. For example, Normando et al. [51] reported that 15 min of daily human interaction, over a 5-week period, increased the amount of time dogs spent at the front of kennels and wagging their tails towards people, compared to a control group; both behaviours are viewed positively by potential adopters [48]. However, we found no consistent change in the level of Positive Active behaviour or time spent at the front on the kennel following DTP participation.

Negative Anxious behaviours (e.g., stressed or vigilant) were most prevalent in kennels and did not significantly decrease following DTP participation. Although socialisation and training can reduce anxiety and stress in dogs [45,46], these are also affected by predisposing genetic factors, environmental factors and early-life experiences [12,20]. There was no relationship between Negative Anxious behaviours at baseline and subsequent time spent in the DTP, consistent with previous findings that displaying fearful behaviours does not predict a longer stay in a shelter [52]. There was a non-significant trend suggesting a reduction in Negative Active behaviours in kennels. Only six of the 24 dogs displayed these behaviours at baseline and this reduced to two dogs following DTP participation. The prevalence of Negative Active behaviours observed at baseline was also positively correlated with subsequent time spent in the DTP, consistent with findings that potential adopters are more sensitive to the presence of negative than positive behaviours [52]. Larger reductions were seen in Negative Active behaviour for dogs remaining in the DTP for longer, indicating that some problematic behaviours can be ameliorated over time [23].

In the training barn, there were high levels of Positive Active behaviours and a moderate level of social interaction with a familiar staff member evident at both time points, and both are judged favourably by people adopting dogs [52,53]. Rescue dogs are often motivated to engage with both familiar and unfamiliar people when given an opportunity [23,48], and previous research indicates that dogs are more engaged and sociable following training sessions [18]. However, we found considerable individual variation in levels of social interaction and no overall increase following DTP participation. Nonetheless, there may be potentially important differences in the quality of these social interactions that our measures did not capture, such as the degree of reciprocity within the dyad, which could also be applied to evaluating the quality of interactions within training sessions [54]. Most dogs (12/17, 70%) also engaged in some Negative Active behaviours and there was no overall reduction following DTP participation. In contrast, Hennessy et al. [23] reported that dogs were calmer following a 3 week in-house DTP and were less likely to jump up or vocalise in response to an unfamiliar person. However, this effect was only evident when a novel moving object was also activated, indicating that a DTP may be beneficial in reducing negative behaviours elicited by more ambiguous, potentially threatening contexts [23].

Subjective staff ratings indicated that there were more Desirable and fewer Undesirable behaviours observed following DTP participation. The dogs were perceived to be more playful, friendly and relaxed, and less excitable, frustrated, noisy, reactive or vigilant, and less likely to pull on the lead or pull towards other people or dogs. Although there was an overall increase in subdued/depressed behaviour, no dogs were rated as displaying this behaviour more than ‘not very often’ following the DTP. There was no change in the perceived prevalence of avoidant and stressed behaviours, suggesting these are more resistant to change or not as effectively addressed within the DTP. Previous findings for shelter-housed dogs using behavioural and subjective rating measures also indicate that stress behaviours remain stable over time [12,50]. However, while four dogs were rated as displaying repetitive, destructive or stressful behaviours ‘very often’ at baseline (either in kennel or barn), none were rated as displaying these more than ‘sometimes’ following the DTP.

Appropriate behaviour towards other dogs is considered an important characteristic for potential adopters [15] but we found no change in the prevalence of Desirable or Undesirable behaviour in the context of passing other dogs. Problematic behaviours in response to encountering other dogs may be particularly challenging to address because these interactions are dynamic and can be unpredictable [55]. Dogs remaining in a shelter for a longer term (>6 months) were rated as more likely to react to other dogs than those with shorter stays (<3 months), but it is unclear if the behaviour was a cause or consequence of remaining at the shelter [15,50]. While daily positive human interaction over a short period has been shown to lead to reduced aggression in rescue dogs, the intervention was specifically aimed at a subpopulation of dogs assessed as exhibiting fearful aggression, and reactions to other dogs were not reported independently of aggressive responses in other contexts [18]. Overall, participation in the DTP increased Desirable behaviours and reduced Undesirable behaviours; however, it may not mitigate all of the effects of underlying anxiety or stress, which can be persistent in relinquished dogs and exacerbated in some contexts [12,50].

Both the behavioural and subjective ratings indicate substantial individual variation in dogs’ coping behaviours in a shelter environment [12]. The positive training outcomes are partially validated by the measures collected at the rehoming centre; for example, staff ratings indicate that dogs were considered more friendly and relaxed, and less likely to pull on their lead during walks and when passing other people and dogs. Similarly, staff perception that the dogs’ demeanour became more relaxed is consistent with the increased time spent in Positive Inactive behaviours during observations in the home kennel. However, while the dogs were rated as more friendly and playful following the DTP, we found no change in Positive Active behaviours or the level of social interaction during the barn observations. These inconsistencies are likely to reflect that the brief video only provided a snapshot of each dog’s behaviour, while the staff ratings allowed a more holistic overview across a wider range of contexts. Overall, the brief observations better approximate the limited time, often less than a minute, that potential adopters initially spend viewing individual dogs [48], but the training and subjective ratings are less time consuming to collect and interpret than quantitative behaviour measures [13]. Moreover, the staff ratings of both Desirable and Undesirable behaviours at baseline were significantly correlated with subsequent time in the DTP, indicating that these ratings were moderately good predictors of adoptability [2].

Monitoring tools can serve multiple purposes within a community service DTP; for example, recording training progress is a simple and efficient measure which can enhance communication with various stakeholders [56]. Within a DTP, training scores allow handlers and staff to assess how well each dog is doing and identify specific training needs, while also providing objective feedback to handlers on their effectiveness as trainers. Records of training progress and recognised awards also contribute to the adoption packs commonly used by rescue organisations to provide further information on individual dogs. Similarly, subjective staff assessments are important in DTPs because handlers and staff are not initially familiar with individual dogs, as is generally recommended for animals engaged in AAIs [29]. Subjective ratings can also inform the focus of individual training plans, for example, to desensitize and address specific behavioural problems such as reactive responses to other dogs [55].

Collectively, these findings indicate that the rescue dogs benefitted from participation in a DTP in terms of training progress, increased levels of relaxed behaviours observed in their home kennels and enhanced subjective ratings of behaviour. However, the selection criteria and availability of suitable dogs for participation in the DTP prevented the inclusion of an appropriate control group, as many dogs spend only a brief period in the rehoming shelter. As a result, it is not possible to fully exclude other factors when interpreting any changes in behaviour beyond the immediate training context. For example, the increase in Positive Inactive behaviour observed in home kennels could reflect habituation to the shelter environment over time. However, activity levels generally remain stable over time in shelters [12,50], and we found no correlation between the amount of change in Positive Inactive behaviours and the total time dogs remained in the DTP. In addition, the subjective ratings indicated a general improvement in the behaviour of almost all of the dogs, while previous research indicates that rates of activity and stereotypical and stress-related behaviours do not generally change over time spent in the shelter context [12,49,50].

Overall, we found no indications that participation in the DTP was detrimental to the dogs’ behaviour or welfare, broadly consistent with previous findings that participation in AAI has no or minimal negative impact on dog welfare if IAHAIO [29,57] guidance is followed [20,28,33]. For DTPs, as for other forms of AAI, appropriate and ongoing assessments, regulated protocols and training procedures should minimise the stress to which dogs are exposed and help to safeguard both people and dogs [25,34]. Identifying the factors shaping the welfare and outcomes of rescue dogs is likely to be challenging because prison-based DTPs are heterogeneous [22]. For example, in the programme evaluated by Hennessy et al. [23], dogs lived and trained with their handlers over a three-week period before becoming available for adoption, while in the current study, the dogs remained at the shelter, participated in the course a few times a week, and the length of participation was primarily determined by whether or not they were rehomed. Given the limited evidence available, it is not clear how the type of training programme (in house or short visits) might impact the efficacy of DTPs in improving outcomes for the dogs involved.

Welfare assessment in AAIs is challenging because of considerable variance in the dogs’ responses, due to individual differences and previous experiences, which may be compounded in DTPs working with relinquished dogs [12,13,34]. Nonetheless, the DTP provided the dogs with a regular routine, enhanced opportunities for socialization and positive reinforcement training [24], which have previously been found to improve the quality of life for dogs in shelters [14,37,38] and likelihood of adoption [15]. Finally, by providing humane education and training skills to the young men, the DTP is likely to have a wider impact on welfare in the community context, albeit one that is difficult to quantify. Trainers demonstrated a substantially increased understanding of the dogs’ physical and emotional needs, felt that their experience in the DTP had positively impacted their attitudes towards and treatment of dogs and also planned to share their learning with family and friends in the future [35].

## 5. Conclusions

While there has been an increased focus on the efficacy of DTPs for human participants [27], there is an equal need to monitor and evaluate the impact on the dogs involved [23,24]. Our results indicate that participation was beneficial for the rescue dogs, in terms of facilitating social enrichment and improvement in training performance. Although behavioural measures indicated a limited impact on dogs outside the training sessions, staff ratings identified general improvement in a range of behaviours across contexts. These positive changes in behaviour and training performance are likely to contribute to improved longer-term outcomes for the dogs. DTPs can be effective in supporting the work of animal rescue organisations by providing positive experiences which benefit both human and dog participants, consistent with a One Health and One Welfare approach [30].

## Figures and Tables

**Table 1:** Overview of behaviours and allocation to categories in video observations and/or subjective rating category. The full behaviour definitions are in Table S1.

Behaviour	Video Observation Category	Subjective Rating Category
Alert	Positive Active	-
Explore	Positive Active	-
Playful/friendly	Positive Active ^a^	Desirable
Play with toy	Positive Active	-
Relaxed	Positive Inactive	Desirable
Resting	Positive Inactive	-
Destructive	Negative Active	Undesirable ^b^
Excited	Negative Active	Undesirable
Frustrated	Negative Active	Undesirable
Reactive	Negative Active	Undesirable
Repetitive	Negative Anxious	Undesirable ^b^
Stressed	Negative Anxious	Undesirable
Vigilant	Negative Anxious	Undesirable
Location	Front of Kennel ^b^	-
Contact/attention	Human Interaction ^a^	-
Noisy		Undesirable
Subdued/Depressed	-	Undesirable
Avoidant	-	Undesirable ^c,d,e^
Pulls on lead	-	Undesirable ^c^
Pulls towards	-	Undesirable ^d,e^

Context-specific behaviours: ^a^ barn only; ^b^ kennel only; ^c^ during walks; ^d^ passing other dogs, and ^e^ interacting with people.

**Table 2:** Median (and inter-quartile range) training task scores across the first three training sessions attended (n = 42 dogs). Training task scores ranged from 0 = no appropriate response to 4 = immediate response (see Section 2.4.1). ^a,b,c^ denote differences identified using Wilcoxon pairwise comparisons (*p* < 0.016); *η*^2^ calculated for pairwise comparison between session 1 and 3.

Training Task	Session 1	Session 2	Session 3	Comparison
Go to bed	2 (1.75–2) ^a^	2 (2–3) ^b^	3 (2–3) ^b^	*χ*^2^ = 44.07, *p* < 0.001, *η*^2^ = 0.24
Door safety	2 (1–2.25) ^a^	2 (2–3) ^b^	3 (2–3) ^b^	*χ*^2^ = 39.57, *p* < 0.001, *η*^2^ = 0.24
Down	2 (1–3) ^a^	2 (2–3) ^b^	3 (3–4) ^b^	*χ*^2^ = 41.69, *p* < 0.001, *η*^2^ = 0.26
Food manners	2 (2–3) ^a^	3 (2–3) ^a,b^	3 (2.75–3.25) ^b^	*χ*^2^ = 33.28, *p* < 0.001, *η*^2^ = 0.19
Greeting	2 (1.75–2) ^a^	3 (2–3) ^b^	3 (2–3) ^b^	*χ*^2^ = 38.97, *p* < 0.001, *η*^2^ = 0.25
Handling	2 (2–3) ^a^	3 (2–3) ^b^	3 (2–4) ^b^	*χ*^2^ = 34.90, *p* < 0.001, *η*^2^ = 0.19
Kennel entry	1 (1–2) ^a^	2 (1–2) ^a,b^	2 (1–3) ^b^	*χ*^2^ = 29.68, *p* < 0.001, *η*^2^ = 0.14
Walk on lead	2 (1–3) ^a^	3 (2–3) ^b^	3 (2–4) ^c^	*χ*^2^ = 46.00, *p* < 0.001, *η*^2^ = 0.30
Walk on lead (distraction)	1 (1–2) ^a^	2 (2–3) ^b^	2 (2–3) ^b^	*χ*^2^ = 40.36, *p* < 0.001, *η*^2^ = 0.26
Leave item	1 (1–2) ^a^	1 (1–3) ^a^	2 (1–3) ^b^	*χ*^2^ = 39.68, *p* < 0.001, *η*^2^ = 0.23
Leave food	2 (1–3) ^a^	2 (2–3) ^b^	3 (2–3) ^b^	*χ*^2^ = 34.20, *p* < 0.001, *η*^2^ = 0.19
Play manners (retrieve)	1 (1–2) ^a^	2 (1–3) ^a,b^	2 (1–3) ^b^	*χ*^2^ = 38.68, *p* < 0.001, *η*^2^ = 0.21
Play manners (tug)	2 (1–2.25) ^a^	2 (1–3) ^a,b^	3 (2–3) ^b^	*χ*^2^ = 34.00, *p* < 0.001, *η*^2^ = 0.16
Recall	2 (2–3) ^a^	3 (2–3) ^b^	3 (3–4) ^b^	*χ*^2^ = 45.17, *p* < 0.001, *η*^2^ = 0.31
Respond to name	2 (2–3) ^a^	3 (2–3) ^b^	3 (3–3) ^b^	*χ*^2^ = 41.70, *p* < 0.001, *η*^2^ = 0.22
Sit	2 (2–3) ^a^	3 (3–3) ^b^	3 (3–4) ^b^	*χ*^2^ = 31.52, *p* < 0.001, *η*^2^ = 0.18
Stand	2 (2–3) ^a^	3 (2–3) ^a,b^	3 (3–4) ^b^	*χ*^2^ = 31.57, *p* < 0.001, *η*^2^ = 0.15
Stay	1 (1–2) ^a^	2 (1–3) ^b^	2 (1–3) ^b^	*χ*^2^ = 43.62, *p* < 0.001, *η*^2^ = 0.27
Wait and recall	1 (1–2) ^a^	2 (1–3) ^b^	2 (2–3) ^b^	*χ*^2^ = 44.04, *p* < 0.001, *η*^2^ = 0.30
Total score (% of maximum possible = 76)	44 (39–53) ^a^	58 (49–68) ^b^	65 (55–75) ^c^	*χ*^2^ = 76.06, *p* < 0.001, *η^2^* = 0.87

**Table 3:** Spearman correlations between total time in weeks spent in the DTP and baseline (pre-DTP) behaviour and overall percentage change scores (n = 17 dogs).

	Measure	Baseline: n Weeks	% Change: n Weeks
Kennel	Positive Active	*r_s_* = −0.05, *p* = 0.86	*r_s_* = 0.17, *p* = 0.51
	Positive Inactive	*r_s_* = −0.21, *p =* 0.41	*r_s_* = 0.10, *p* = 0.70
	Negative Active	***r_s_*** **= 0.57 *p* = 0.017**	***r_s_*** **=** **−0.57, *p* = 0.016**
	Negative Anxious	*r_s_* = −0.28, *p* = 0.27	*r_s_* = −0.10, *p* = 0.71
	Front of Kennel	*r_s_* = −0.05, *p* = 0.86	*r_s_* = −0.08, *p* = 0.76
Barn	Positive Active	*r_s_* = 0.05, *p* = 0.86	*r_s_* = −0.13, *p* = 0.62
	Positive Inactive	*r_s_* = −0.21, *p* = 0.43	*r_s_* = 0.21, *p* = 0.43
	Negative Active	*r_s_* = −0.05, *p* = 0.86	*r_s_* = 0.14, *p* = 0.96
	Negative Anxious	*r_s_* = −0.18, *p* = 0.50	*r_s_* = −0.11, *p* = 0.66
	Human Interaction	*r_s_* = 0.13, *p* = 0.61	*r_s_* = 0.04, *p* = 0.88

Values in bold *p* < 0.05.

**Table 4:** Median percentage scores (inter-quartile range) for behavioural categories rated by staff at baseline (pre-DTP) and the final ratings following DTP participation (n = 20 dogs). Rating scores ranged from 1 = not at all to 5 = very often (see Section 2.4.3).

Behaviour	Baseline	Final	Comparison
Playful/friendly	60 (55–69)	75 (66–80)	*z* = 3.09, ***p* = 0.002**, *η*^2^ = 0.24
Relaxed	50 (40–55)	60 (51–65)	*z* = 2.94, ***p* = 0.003**, *η*^2^ = 0.22
All Desirable	57 (48–63)	66 (58–74)	*z* = 3.28, ***p* = 0.003**, *η*^2^ = 0.27
Avoidant ^b,c,d^	47 (40–60)	47 (40–60)	*z* = 0.37, *p* = 0.71, *η*^2^ = 0.003
Destructive ^a^	20 (20–50)	40 (20–40)	*z* = 0.61, *p* = 0.54, *η*^2^ = 0.01
Excitable	65 (60–78)	60 (56–60)	*z* = 2.43, ***p* = 0.015**, *η*^2^ = 0.15
Frustrated	65 (55–83)	55 (46–60)	*z* = 2.62, ***p* = 0.009**, *η*^2^ = 0.17
Noisy	63 (48–70)	55 (45–60)	*z* = 3.00, ***p* = 0.003**, *η*^2^ = 0.22
Pull lead ^b^	60 (60–80)	60 (45–60)	*z* = 2.27, ***p* = 0.023**, *η*^2^ = 0.13
Pull towards ^c,d^	60 (50–78)	60 (43–60)	*z* = 2.50, ***p =* 0.012**, *η*^2^ = 0.16
Reactive	45 (40–60)	50 (36–53)	*z* = 2.07, ***p* = 0.038**, *η*^2^ = 0.11
Repetitive ^a^	20 (20–40)	40 (20–40)	*z* = 0.28, *p* = 0.78, *η*^2^ = 0.002
Stressed	60 (50–64)	60 (45–60)	*z* = 1.66, *p* = 0.10, *η*^2^ = 0.07
Subdued/depressed	25 (20–34)	40 (25–40)	*z* = 2.01, ***p* = 0.044**, *η*^2^ = 0.10
Vigilant	60 (45–74)	43 (40–50)	*z* = 3.11, ***p* = 0.002**, *η*^2^ = 0.24
All Undesirable	54 (48–61)	49 (43–54)	*z* = 2.89, ***p* = 0.004**, *η*^2^ = 0.21

Context specific behaviours: ^a^ kennel only, ^b^ during walks, ^c^ passing other dogs, and ^d^ interacting with people. Values in bold *p* < 0.05.

## Data Availability

The original data presented in the study are openly available in the Stirling Online Research Repository (STORRE) at http://hdl.handle.net/11667/230 accessed on 6 May 2024.

## Supplementary Materials

The following supporting information can be downloaded at: https://www.mdpi.com/article/10.3390/ani14111530/s1, Table S1: Descriptions of behaviours coded in video observations and scored by staff using the subjective rating scale. References [39,44,50,58,59,60,61] are cited in the supplementary materials.
