# Supplementary material for: Participation in a Prison-Based Training Programme Is Beneficial for Rescue Dogs
Source: Animals (Basel). 2024 May 22;14(11):1530. doi: 10.3390/ani14111530 (PMC11171100; doi:10.3390/ani14111530)
Supplement: Supplementary file 1 [file animals-14-01530-s001.zip › animals-2974553-supplementary.pdf]

Leonardi, Vick & Buchanan-Smith (Animals 2024)

Participation in a prison-based training programme is beneficial for rescue dogs

Table S1. Descriptions of behaviours coded in video observations and scored by staff using the subjective rating scale.

Behaviour	Description	Video Observation Category	Subjective Rating Category
Alert	Visually monitoring stimulus in environment, without signs of excessive tension or freezing.	Positive Active	-
Explore	Purposeful investigation of environment, sniffing or following scent, may include locomotion.	Positive Active	-
Playful/ friendly	Attentive and initiates interaction with people or dogs, includes polite approach, invitation to play (e.g. toy present, rollover or bow) and engagement in joint play, accompanied by tail wagging or wiggly body movements; may solicit physical contact and display affection, e.g. by leaning against or affectionate licking. May offer learned behaviours (e.g. offer paw).	Positive Active[a]	Desirable
Play with toy	Includes elements of predatory behaviour, including stiff-legged jumping, grabbing, chewing, head shaking.	Positive Active	-
Relaxed	Movements are calm with no signs of tension, with soft eyes, relaxed ear posture and tail position, may include stretching. [Desirable category: includes normal patterns of eating, drinking, resting, sleeping and investigating].	Positive Inactive	Desirable
Resting	Reclining in ventral or lateral position, eyes open or closed.	Positive Inactive	Desirable
Destructive	Chews, bites, gnaws, shreds, digs or scratches beds, toys, blankets or other non-food items.	Negative Active	Undesirable [b]
Excited	Easily aroused and impatient, unable to settle, can become over-aroused; quick movements and rapid changes in behaviour, may include jumping, spinning, whining or barking, teeth chattering, paw treading, or attempts to leave; may be re-directed at objects e.g. snatching or ragging toys.	Negative Active	Undesirable
Frustrated	Inability to calm or settle, lack of concentration, reaching or lunging forward, scrabbling at windows, doors or walls. May include vocalisations (e.g. whine, whinge or bark). May redirect frustrations onto person by attention-seeking or contact (e.g. jumping up, muzzle punch, or scratching), rapidly offering alternative behaviours, or trying to get rewards (e.g. grabbing, or mouthing at pockets), or onto objects, such as frantically ragging toy or lead.	Negative Active	Undesirable

Reactive	Responds to stimulus in environment (e.g. people, dogs, or noise); may freeze or move towards stimulus, with tense posture, head high and forward, tail raised, hard stare, hackles up; may include lunging, standing on rear legs, jumping up or scratching at walls/windows in kennels, and be accompanied by barking, growling, baring teeth, snarling, cheek puffing or salivation.	Negative Active	Undesirable
Repetitive	Performs behaviour in repetitive or compulsive manner, such as pacing, spinning, tail chasing, licking or chewing body parts; may include bouncing on walls, or shadow chasing.	Negative Anxious	Undesirable [b]
Stressed	Tense posture, ears pinned back, eyes narrowed, mouth pulled back, may include lip licking, yawning, excessive panting or scratching; cautious and signs of avoidance, may react to stimulus by flinching, turning away, trembling, tail tucked, cowering or seeking cover, raising paw, performing urogenital check, or rolling onto back.	Negative Anxious	Undesirable
Vigilant	Alert with intense focus on stimulus in environment, unable to concentrate on other stimuli, accompanied by signs of anxiety, tension or freezing, may include hard stare or rapid eye and ear (pricking) movements, frantic air scenting.	Negative Anxious	Undesirable
Location	Dog is located in the front half of their kennel.	Location [b]	
Contact/ attention	Performs behaviour when in physical contact, making eye contact, or oriented towards and attending to a person.	Human Interaction [a]	
Noisy	Any form of vocalisation: barking, whining or howling	-	Undesirable
Subdued/ depressed	Lacks interest, may appear indifferent to stimulus in the environment. May include sleeping too much or too little, no motivation or interest in activities or social contact, unresponsive. Slumped posture, dull eyes and lack of attention.	-	Undesirable
Avoidant	Avoids stimulus in the environment, such as attempts to approach, reach towards, initiate contact or touch by person or dogs. Includes avoiding eye contact, ducking head, flinching, tail tucked, head or body turn, move away, cowering, seeking cover or hiding (e.g. behind familiar person). May include lie down, freeze or roll over when unable to move away.	-	Undesirable [c,d,e]
Pulls on lead	Putting force or pressure on lead, or throwing weight to influence direction. If unable to move may scrabble or fight constraints of lead (e.g. with a fast change in direction).	-	Undesirable [c]
Pulls towards	Alert and moves towards stimulus, excitable attempts to approach and investigate, which may become frustrated or frantic; pressure on lead and may scratch or scrabble towards stimulus.	-	Undesirable [d,e]

Context specific: [a] Barn only; [b] Kennel only; [c] During walks; [d] Passing other dogs; [e] Interacting with people.
References [39,44,50,58–61]
